# Coronary CT angiography for preoperative evaluation of non-cardiac surgery in patients with thoracic tumors: preliminary exploratory analysis in a retrospective cohort

**DOI:** 10.1186/s13019-022-02096-y

**Published:** 2023-03-20

**Authors:** Meng Liao, Mingyue Tang, Xu Cao, Gao Liang, Mingguo Xie, Peng Zhou

**Affiliations:** 1 School of Medical and Life Sciences, Chengdu University of Traditional Chinese Medicine, Chengdu, 610072 China; 2 Department of Radiology, Hospital of Chengdu University of Traditional Chinese Medicine, Chengdu, 610072 China; 3 Department of Radiology, Sichuan Cancer Hospital and Institute, Sichuan Cancer Center, School of Medicine, University of Electronic Science and Technology of China, Chengdu, 610041 China

## Abstract

**Purpose:**

Noninvasive coronary CT angiography (CCTA) was used to retrospectively analyze the characteristics of coronary artery disease (CAD) in patients with thoracic tumors and the impact of the results on clinical surgery decision-making, thus increasing the understanding of perioperative cardiac risk evaluation.

**Method:**

A total of 779 patients (age 68.6 ± 6.6 years) with thoracic tumor (lung, esophageal, and mediastinal tumor) scheduled for non-cardiac surgery were retrospectively enrolled. Patients were divided into two groups: accepted or canceled surgery. Clinical data and CCTA results were compared between the two groups, and multivariate logistic regression analysis was performed to determine predictors of the events of cancellations of scheduled surgeries.

**Results:**

634 patients (81.4%) had non-significant CAD and 145 patients (18.6%) had significant CAD. Single‑, 2‑, and 3‑ vessel disease was found in 173 (22.2%), 93 (11.9%) and 50 (6.4%) patients, respectively. 500 (64.2%), 96 (12.3%), 96 (12.3%), 56 (7.2%) and 31 (4.0%) patients were rated as CACS 0, 1–99, 100–399, 400–999 and > 1000, respectively. Cancellations of scheduled procedures continue to increase based on the severity of the stenosis and the number of major coronary artery stenosis. The degree of stenosis and the number of vascular stenosis were independent predictors of cancelling scheduled surgery.

**Conclusions:**

For patients with thoracic tumors scheduled for non-cardiac surgery, the results suggested by CCTA significantly influenced surgery planning and facilitated to reduce perioperative cardiovascular events.

## Introduction

Surgery is one of the most common treatments for patients with thoracic tumors, such as lung, esophageal and mediastinal tumors. These patients may have occult CAD with inconspicuous and atypical clinical symptoms, thus has not been diagnosed. Major adverse cardiac events (MACE) that occur after non-cardiac surgery are usually associated with prior CAD. Risk assessment of perioperative cardiovascular events is important for clinical surgical planning. CCTA is a noninvasive examination with high sensitivity and specificity in the detection or exclusion of CAD [1]. Although current guidelines do not include CCTA as a routine preoperative examination for patients undergoing non-cardiac surgery [2], CCTA is increasingly being used for preoperative screening as a reliable method of diagnosing CAD in clinical practice. Previous research has shown that the severity and extent of CAD in CCTA in non-cardiac surgery patients are associated with perioperative MACE [3]. However, in clinical practice, little attention has been paid to whether coronary stenosis and calcification on CCTA has an impact on surgical planning. Therefore, we retrospectively analyzed the characteristics of CAD in patients with thoracic tumors scheduled for non-cardiac surgery and the impact of CCTA results on scheduled surgery to increase our understanding of perioperative management.

## Materials and methods

### Study population

**Fig. 1:**
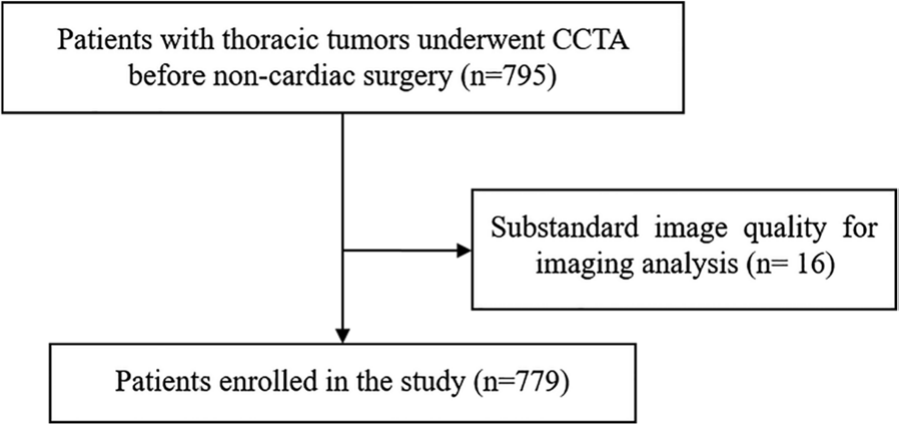
Flow diagram of the study patients

The study was approved by a local institutional review committee. Due to the retrospective design of this study, all subjects waived informed consent. From January 2015 to June 2019, we enrolled a total of 795 patients with non-cardiovascular thoracic tumor surgery who underwent preoperative CCTA for screening of CAD. Exclusion criteria in this study were: left ventricular ejection fraction < 40%, renal insufficiency (glomerular filtration rate < 30 ml/min/1.7 m^2^), severe heart failure, severe arrhythmia, iodine contrast agent allergy, and substandard image quality for imaging analysis. Finally, we recruited 779 patients (Fig. 1) with thoracic tumor, among them, there were 289 cases of lung tumor, 470 cases of esophagus tumor and 20 cases of mediastinal tumor. The preoperative complications of cardiovascular diseases included 248 patients with hypertension, 234 patients with hyperlipidemia, 50 patients with confirmed CAD, and 92 patients with the positive electrocardiogram (ECG).

### Scan protocols

CCTA of all patients was performed on a Philips Brilliance 256-layer scanner using a retrospective electrocardiogram gated mode. Scanning scope ranged from tracheal carina to 2 cm below the apex of the heart. First, plain CT scan was used for quantitative measurement of coronary artery calcification scores. Imaging parameters: prospective gated, triggered at 75% R-R interval, tube voltage 120 kV, tube current 550 mAs, slice thickness 2.5 mm, reconstruction interval 2.5 mm and rotation time 0.27 s. A 20-gauge needle with double tube high-pressure Syringe (Boluspro, Philips Healthcare, Cleveland, Ohio, USA) Contrast agent (Ultravist 370, Bayer Healthcare, Berlin, Germany) was injected through the cubital vein 60–80 mL at a flow rate of 5.0 mL/s. After injection, 30 mL of normal saline was injected at the same rate. The ascending aorta was set as the region of interest, and the trigger threshold was set at 100–120HU. After reaching the threshold, the patient was asked to hold his breath, and the scan was automatically triggered after 6 s. Scanning parameters: tube voltage 100 kV, tube current 400–500 mAs, detector collimator 128 × 0.625 mm, rotation time 0.27 s, pitch 0.18, standard reconstruction(iDose^4^, level 5), reconstructed slice thickness 0.9 mm, reconstructed interval 0.45 mm.

### Data analysis

**Fig. 2:**
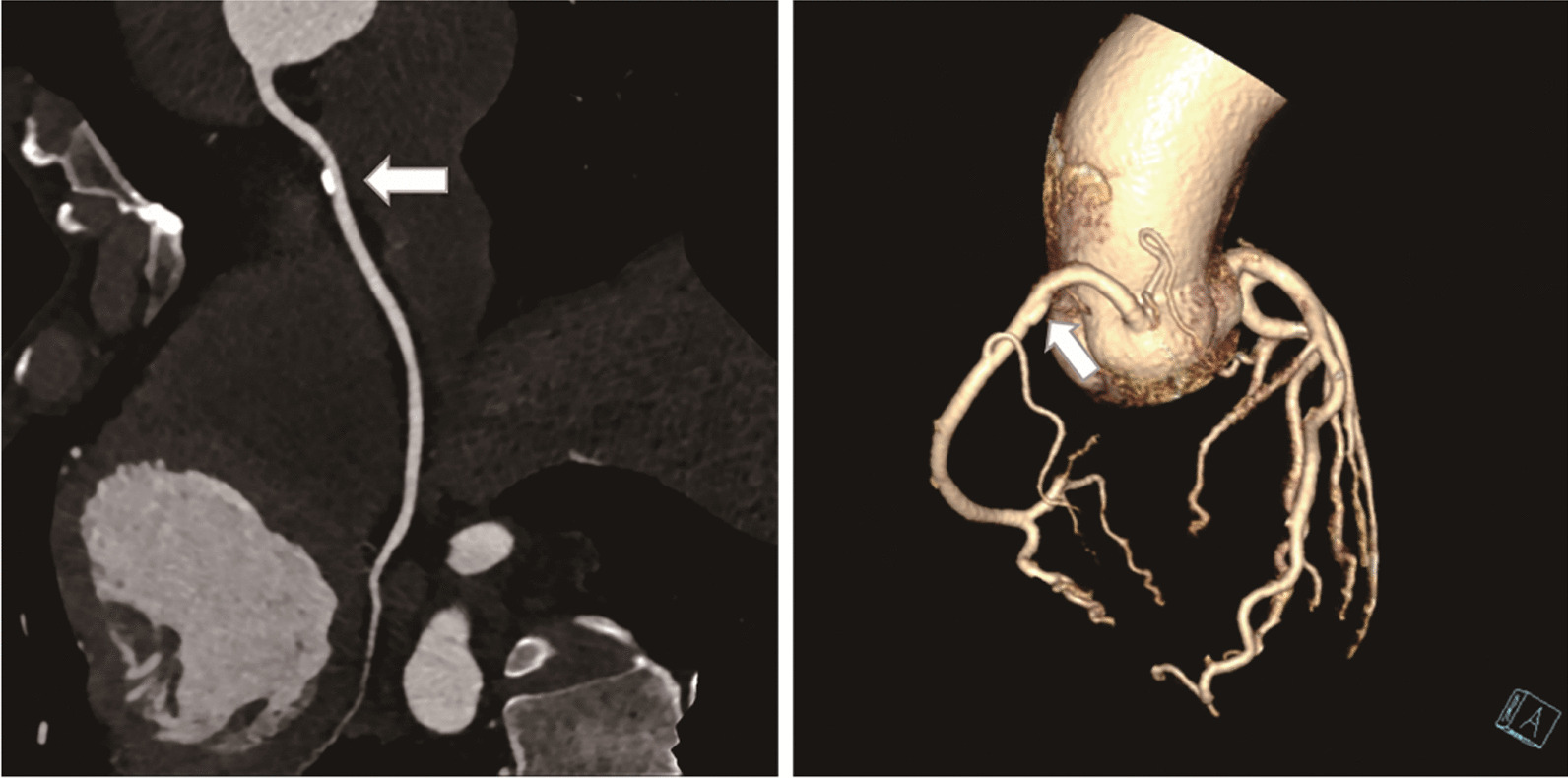
An example of mild stenosis in 65‑year-old asymptomatic man with esophageal cancer. Localized calcified plaque (arrow) with mild stenosis in the proximal segment of right coronary artery (RCA). Abbreviations: RCA, right coronary artery

**Fig. 3:**
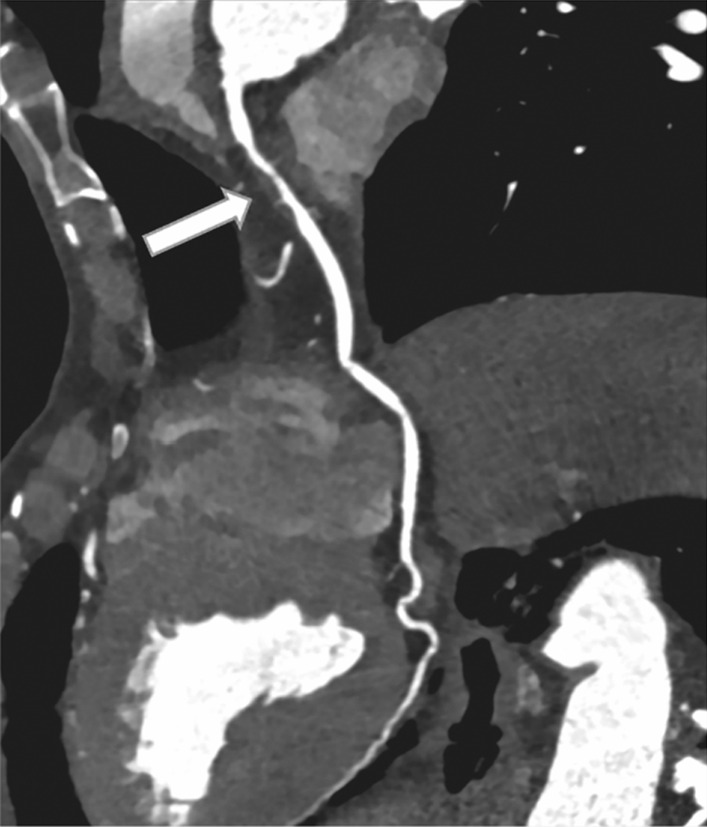
An example of moderate stenosis in 74‑year-old asymptomatic man, non‑calcified plaque (arrow) with moderate stenosis in the proximal segment of left anterior descending coronary artery (LAD). Abbreviations: LAD, left anterior descending coronary artery

**Fig. 4:**
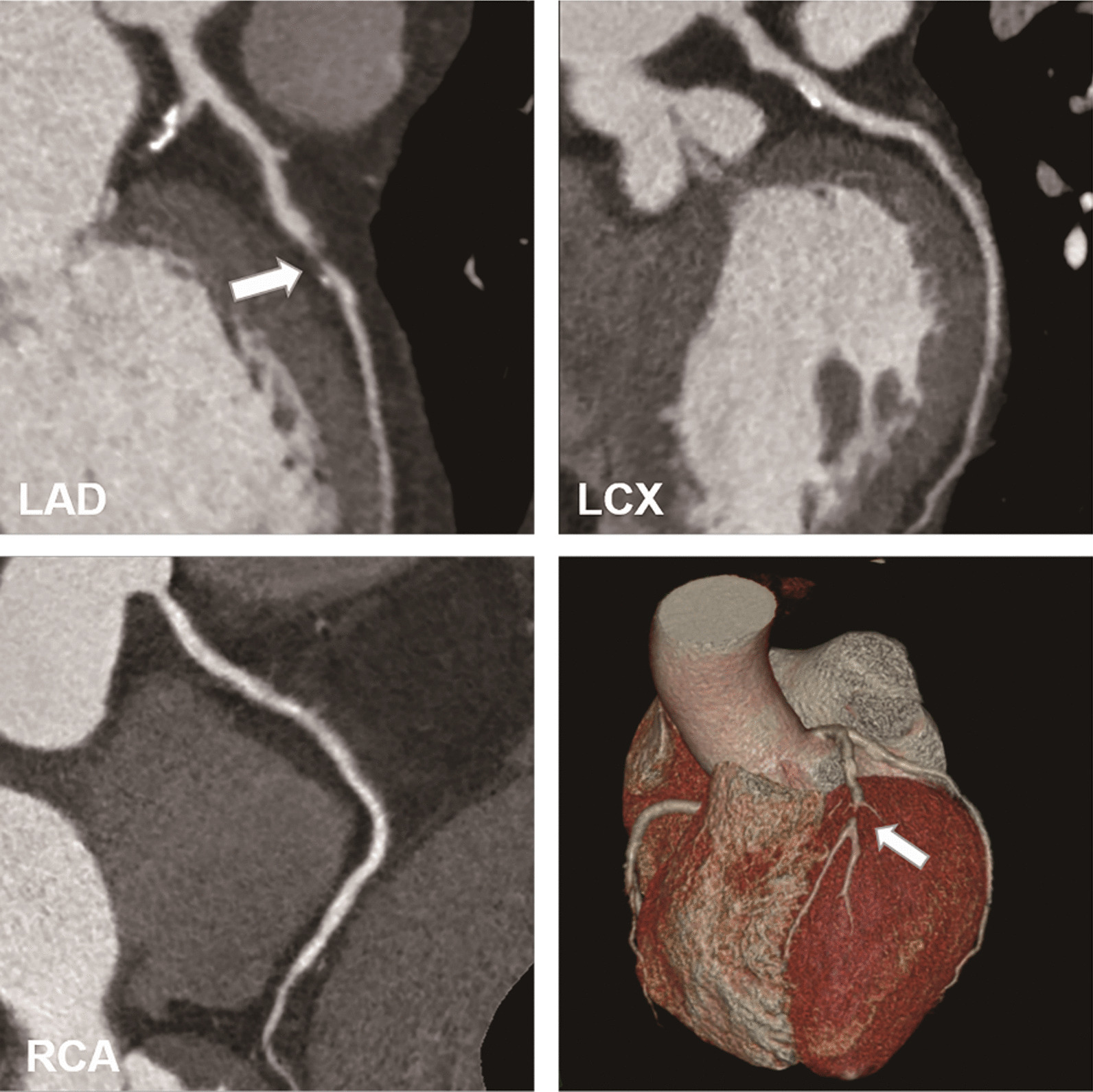
An example of severe stenosis in 54‑year-old man with chest pain and positive ECG analysis. Mixed plaque (arrow) with severe stenosis in the middle segment of left anterior descending coronary artery (LAD). Localized calcified plaque in the left circumflex coronary artery (LCX) with no luminal stenosis. The right coronary artery (RCA) is normal appearing. Abbreviations: LAD, left anterior descending coronary artery; LCX, left circumflex coronary artery; RCA, right coronary artery

Two experienced radiologists independently reviewed each CT scan on a dedicated workstation (Extended Brilliance Workspace Version 4.0; Philips Healthcare). If no consensus can be reached, a third expert is consulted to make the final diagnosis. Image post-processing methods used to evaluate coronary stenosis and calcification include maximum density projection, multiplane reconstruction, curved surface reconstruction, and volume reconstruction. 1-, 2-, or 3-vessel disease was defined according to the number of epicardial arterial stenosis. In patients with multi-vessel disease, the most severe coronary artery stenosis was considered the study subject. The degree of coronary artery stenosis was classified as normal appearing, mild (< 50%, Fig. 2), moderate (50%-75%, Fig. 3), and severe (≥ 75%, Fig. 4) stenosis. Among them, normal appearing and mild stenosis were non-obstructive stenosis, and moderate and severe stenosis were obstructive stenosis. Coronary artery calcification score (CACS) was obtained by smartscore software and divided into 5 groups, namely 0, 1–99, 100–399, 400–999 and > 1000.

Electronic medical records were reviewed retrospectively to analyze patients' clinical data, including coronary risk factors such as hypertension, diabetes, hyperlipidemia, smoking, stroke, and related clinical decisions (Table 1). In preoperative routine ECG examination, ST segment analysis is considered positive if ST segment horizontal or down-sloping depression ≥ 1 mm occurs in 2 or more consecutive leads. The impact of CCTA results on clinical decisions was determined after a multidisciplinary consultation, that is, whether surgery was delayed or canceled due to severe CAD.

**Table 1:** Patient characteristics

	Accept surgery n = 484	Cancel surgery n = 295	*p* value
Age	68.7 ± 6.4	68.4 ± 6.8	0.542
Male/Female	368/116	230/65	0.536
BMI	22.8 ± 3.1	22.3 ± 3.2	0.032
HR (beats/min)	73.2 ± 31.1	74.3 ± 14.7	0.585
ECG Positive/Negative	46/434	46/247	0.011
LEVF	66.8 ± 7.0	65.9 ± 8.1	0.120
Smoking/Non-smoking	229/255	158/137	0.091
Hypertension/ Non-hypertension	157/328	91/203	0.680
Hyperlipidemia/Non-hyperlipidemia	148/336	86/209	0.674
Stroke/Non-stroke	12/472	13/282	0.139
Diabetes/Non-Diabetes	38/446	29/265	0.357

Abbreviations: BMI, Body Mass Index; HR, heart rate; ECG, electrocardiogram; LECF, Left ventricular ejection fraction

### Statistical analysis

SPSS Version 23.0 and Graph Pad Prism Version 6.0 were used for statistical analysis. Continuous data are expressed as mean ± standard deviation, while nominal variables are expressed as frequency and percentage. The t test was used for measurement data, and the chi-square test or Fisher's exact test was used for counting data. Univariate and multivariate logistic regression analyses were performed to assess which parameters were independently associated with surgical decision making in patients with thoracic tumor. A *p* value < 0.1 in the univariate analyses were introduced to further multivariate analysis. A double-tailed *p* < 0.05 was considered statistically significant.

## Results

### Patient Characteristics

Cardiac CT scanning was successfully performed in all 779 patients, whose age ranged from 41 to 89 years (mean 68.6 ± 6.6 years). Baseline characteristics of subjects in our study are shown in Table 1. No ECG was performed in 6 patients. In 15 patients BMI data was lacking and in 1 patient diabetes was unknown. Among the 779 patients, 145 patients with significant coronary artery stenosis, 12 underwent invasive coronary angiography (ICA) and 1 underwent coronary intervention after coronary CTA. These patients underwent coronary artery treatment followed by non-cardiac surgery.

### The effect of coronary artery CTA on the planning of non-cardiac surgery

Among CAD patients, 55, 28, and 48 patients with mild, moderate, and severe cases gave up surgery, respectively. Table 2 shows the coronary categories as determined by CT. In total, 634 (81.4%) patients had non-significant CAD and 145 (18.6%) patients had significant CAD. Of the patients with non-significant CAD, 463 (59.4%) patients were normal and 171 (22.0%) patients showed mild stenosis. Of the patients with significant CAD, 71 (9.1%) patients had moderate stenosis and 74 (9.5%) patients had severe stenosis. In addition, for stenosis of CAD, 1-, 2-, and 3- vessel disease was found in 173 (22.2%), 93 (11.9%), and 50 (6.4%) patients, respectively (Table 2); and 143 (18.4%) patients showed multi-vessel disease (≥ 2 branches). In addition, scheduled surgery was cancelled in 19 (11.0%), 28 (30.1%) and 19 (38.0%) patients with 1-, 2-, and 3- vessel disease, respectively. In the grading of CACS, 500 (64.2%), 96 (12.3%), 96 (12.3%), 56 (7.2%) and 31 (4.0%) patients were rated as 0, 1–99, 100–399, 400–999 and > 1000, respectively (Table 3). Univariate and multivariate logistic regression analysis showed that, the number and degree of vascular stenosis were independently correlated with the cancelation of surgery (Table 4).

**Table 2:** Degree of coronary artery stenosis and the events of abandoned surgery for the reason of cardiac risk

	Frequency (n, %)	Event (n, %)	*p* value
Non-significant stenosis	634		< 0.001
Normal appearing	463 (59.4%)	0 (0)	
Mild stenosis	171 (22.0%)	0 (0)	
Significant stenosis	145		
Moderate stenosis	71 (9.1%)	24 (33.8%)	
Severe stenosis	74 (9.5%)	42 (56.8%)	
Number of major epicardial coronary artery stenosis			
Normal appearing	463 (59.4%)	0(0)	
1‑vessel disease	173 (22.2%)	19 (11.0%)	0.001^*^
2‑vessel disease	93 (11.9%)	28 (30.1%)	
3‑vessel disease	50 (6.4%)	19 (38.0%)	
Multi-vessel disease	143 (18.4%)	47 (32.9%)	< 0.001^**^

Abbreviations: CAD coronary artery disease

^*^Event compared among 1, 2 and 3-vessel disease

^**^Event compared between 1-vessel disease

**Table 3:** Coronary artery calcification score and the events of abandoned surgery for the reason of cardiac risk

	Frequency (n, %)	Event (n, %)	*p* value
0	500 (64.2%)	0 (0)	< 0.001
1–99	96 (12.3%)	0 (0)	
100–399	96 (12.3%)	27 (28.1%)	
400–999	56 (7.2%)	24 (42.9%)	
> 1000	31 (4.0%)	15 (48.4%)

**Table 4:** Univariate and multifactorial logistic regression analysis of the events of abandoned surgery

Variables	Univariate analysis OR (95% CI)	*p* value	Multivariate analysis OR (95% CI)	*p* value
BMI	1.053 (1.004–1.103)	0.032	1.047 (0.996–1.100)	0.070
ECG	0.569 (0.367–0.881)	0.012	0.777 (0.487–1.242)	0.292
HR	0.998 (0.993–1.004)	0.594		
LEVF	1.016 (0.996–1.037)	0.124		
Number of vascular stenosis	0.830 (0.713–0.967)	0.017	1.365 (1.001–1.863)	0.049
Degree of stenosis	0.749 (0.647–0.866)	< 0.001	0.671 (0.504–0.894)	0.006
CACS	0.999 (0.999–1.000)	0.001	1.000 (0.999–1.000)	0.082
Smoking	0.773 (0.578–1.035)	0.084	0.833 (0.612–1.133)	0.244
Hypertension	1.076 (0.788–1.470)	0.644		
Hyperlipidemia	1.070 (0.780–1.470)	0.674		
Diabetes	0.779 (0.469–1.292)	0.333		
Stroke	0.551 (0.248–1.224)	0.143		

Abbreviations: BMI, Body Mass Index; ECG, electrocardiogram; HR, heart rate; LECF, Left ventricular ejection fraction; CACS, Coronary artery calcification score

According to the electronic hospitalization records, during the postoperative hospitalization, 1 patient (severe stenosis; 2-vessel disease) had non-fatal myocardial infarction, 2 patients died of cardiac shock, and the rest had no MACE records.

## Discussion

The main findings of this study were that the number and degree of vascular stenosis suggested by preoperative CCTA in patients with thoracic tumor was independently associated with the decision to cancel surgery; surgery cancellations increased as the number or extent of the stenosis rise.

Cardiovascular disease is also the leading cause of death for tumor patients, there are common risk factors between them two. Vascular endothelial damage or arterial thrombosis caused by anti-tumor treatment may increase the risk of cardiovascular disease [4]. Tumor patients at higher risk of CAD, while clinical manifestations are atypical, for example, chest pain and dyspnea less seen in tumor patients [5]. Therefore, tumor patients may have a potential risk of CAD, which needs to be paid great attention. CAD can affect or limit tumor treatment. Surgery is a common treatment method for patients with lung tumor, esophageal tumor, or mediastinal tumor, which has certain requirements for the circulatory function of tumor patients. Moreover, type of surgery is related to the cardiac risk. As a high-risk operation, the MACE risk of thoracic surgery is ≥ 5% [6]. Surgical stress leads to inflammation and hypercoagulability, triggering plaque instability or rupture, and subsequent thrombosis, which accounts for 50% of perioperative acute coronary events [7, 8]. Therefore, CAD will limit the feasibility of surgery.

Perioperative MACE was defined as non-fatal stroke, non-fatal myocardial infarction, congestive heart failure, and cardiogenic death that occurred within 30 days after surgery. Worldwide, more than 300 million patients undergo non-cardiac surgery each year [9]. Cardiovascular complications are one of the major causes of MACE in patients undergoing non-cardiac surgery. As the tumor patients with cardiovascular disease undergoing non-cardiac surgery continue to increase, the incidence of perioperative MACE is also increased, which seriously affects the safety of surgery and the management of postoperative complications. Therefore, preoperative risk assessment of the cardiovascular event is of great significance for tumor patients.

CCTA is a non-invasive examination for the assessment of CAD. It can clearly display the type and composition of plaque and accurately evaluate the extent and degree of coronary artery stenosis [10, 11]. In contrast to invasive coronary angiography (ICA), CCTA can show plaques remodeling outward without lumen narrowing [12]. With relatively high sensitivity and specificity, low cost and low radiation, CCTA has become the preferred method of noninvasive examination for diagnosis of CAD [10]. The addition of an appropriate CCTA to enhanced CT in patients with thoracic tumors does not significantly increase radiation exposure or contrast material administration, and providing a practical improvement in cardiovascular risk stratification in these patients [13].

Patients with severe stenosis can be improved by revascularization, while patients with mild or moderate stenosis can be treated with medication [14]. Moreover, patients who were first assessed as inoperable by CCTA may regain the opportunity of surgery after the relevant treatment. Considering the increased heart disease progression or surgical risk, scheduled surgery of tumor patients with CAD may be delayed or cancelled. Therefore, CCTA examination before developing a treatment plan can indicate whether surgery can be performed as scheduled or should be postponed after CAD intervention or abandoned. Even though the current guidelines, CCTA has not been incorporated into the preoperative routine examination, but CCTA as a noninvasive method can be encouraged to performed on tumor patients preoperatively, if the results have a potential influence on the management of patients [2, 15, 16]. The appropriate indication for coronary CTA as part of preoperative evaluation is not specified in current European Society of Cardiology or American College of Cardiology/American Heart Association guidelines, mainly due to insufficient data on coronary CTA in preoperative risk stratification [1], which should be investigated in future research efforts.

Exercise ECG test, stress echocardiography and stress myocardial perfusion imaging are recommended for the screening of CAD [17], but stress test is not suitable for patients with poor general conditions or with contraindications. For non-cardiovascular surgery patients, ICA is not routinely recommended for risk stratification, but ICA and revascularization are recommended before high-risk surgery or accompanied with severe stress ischemia. In this study, 18.6% of patients were assessed as significant CAD by CCTA, but most patients did not undergo ICA. A study [18] suggested that coronary CTA and ICA are equally effective in assessing long-term risk in patients with non-ST-elevation acute coronary syndrome. Although severe calcification may affect the judgment of the degree of luminal stenosis [19], in the clinical practice of non-invasive screening of CAD before surgery for tumor patients, it is more concerned about how to screen out patients who are not suitable for surgery, rather than over-diagnosis.

In our study, with the increase of degree of stenosis, patients gave up surgery has increased because of CCTA results. Some patients with mild coronary artery stenosis experienced plaque rupture leading to fatal cardiovascular events [20], thus we included patients with all grades of stenosis, not just significant stenosis. Patients with multi-vessel disease were more likely to forgo surgery for cardiovascular reasons than patients with single-vessel disease.

A previous study suggested that the more extensive coronary artery calcification was associated with a higher incidence of coronary artery events, which was inconclusive [21]. But recent research suggests that calcification can predict risk of cardiovascular events and death [22–24]. A study [22] of 25,253 asymptomatic patients with long-term follow-up concluded that CACS was an independent predictor of all-cause mortality, the mortality risks of 11–100, 101–299, 300–399, 400–699, 700–999, and > 1000 scores with CACS were approximately 2.2, 4.5, 6.4, 9.2, 10.4, and 12.5 times of those with CACS 0, respectively. Coronary artery calcium scans are recommended as a class IIa in the 2019 ACC/AHA guidelines for people at intermediate risk [25]. People with CACS of zero had a lower incidence of CACS progression or risk of coronary artery disease during the 5-year warranty period [26, 27]. In our study, the number of non-stenotic and non-calcified patients was not equal, possibly because some patients only had non-calcified plaques. The probability of abandoning surgery by CCTA results was significantly different among groups with different calcification scores. However, in multivariate logistic regression analysis, CACS cannot be considered as an independent factor influencing surgical decision making.

A meta-analysis [3] showed that the risk of perioperative MACE was strongly correlated with the extent and severity of coronary artery stenosis indicated by CCTA, with a greater risk of obstructive stenosis and multi-vessel disease; there was also a certain correlation between CACS and the incidence of MACE during the perioperative period (CACS ≥ 100, ≥ 400, ≥ 1000 were compared with CACS < 100, < 400, < 1000 respectively). In this study, of 484 patients who underwent surgery, only 1 had perioperative MACE (non-fatal myocardial infarction) during hospitalization, with an incidence of 0.21%, significantly lower than reported [28, 29]. It can be said that CCTA evaluation can effectively reduce the incidence of cardiovascular events. In addition to providing coronary artery stenosis and plaque information, CCTA can also obtain a series of hemodynamic indicators by combining advanced computational fluid dynamics methods, which were not analyzed in this paper. As a noninvasive and effective visualization tool, CCTA can provide preliminary coronary risk information and reduce cardiovascular complications by excluding some patients who are not suitable for surgery. Meanwhile, it may also exclude some patients who require surgery. The benefits to patients need to be further studied.

### Study limitations

The limitations of this study are as follows. First, due to retrospective study, we could only include perioperative MACE during hospitalization. We cannot fully assess the outcome of the patients, and CCTA cannot currently be recommended as a routine test for preoperative cardiac risk stratification in patients undergoing noncardiac surgery. The second, this is a single-center retrospective study, which may have a certain center-specific bias, and a larger cohort multi-center study should be conducted in the future to investigate the association between CCTA and cardiac risk. The third, CACS for preoperative cardiac risk assessment needs further study.

## Conclusion

For patients with thoracic tumors scheduled for non-cardiac surgery, preoperative CCTA characterizes coronary artery stenosis and calcification to facilitate detecting CAD and risk stratification, thereby influencing clinical surgery decisions.

## Data Availability

The datasets used and/or analysed during the current study are available from the corresponding author on reasonable request.

## References

[1] Hwang JW, Kim EK, Yang JH, Chang SA, Song YB, Hahn JY, Choi SH, Gwon HC, Lee SH, Kim SM, et al. Assessment of perioperative cardiac risk of patients undergoing noncardiac surgery using coronary computed tomographic angiography. Circul Cardiovas Imaging. 2015;8(3). 10.1161/CIRCIMAGING.114.002582 25711275

[2] Arnett DK, Blumenthal RS, Albert MA, Buroker AB, Goldberger ZD, Hahn EJ, Himmelfarb CD, Khera A, Lloyd-Jones D, McEvoy JW (2019). 2019 ACC/AHA guideline on the primary prevention of cardiovascular disease: executive summary: a report of the American College of Cardiology/American Heart Association Task Force on Clinical Practice Guidelines. Circulation.

[3] Koshy AN, Ha FJ, Gow PJ, Han HC, Amirul-Islam FM, Lim HS, Teh AW, Farouque O (2019). Computed tomographic coronary angiography in risk stratification prior to non-cardiac surgery: a systematic review and meta-analysis. Heart (British Cardiac Society).

[4] Nohria A, Groarke JD (2019). Management of acute coronary syndromes in patients with cancer: room for improvement. Eur Heart J.

[5] Guha A, Dey AK, Jneid H, Addison D (2019). Acute coronary syndromes in cancer patients. Eur Heart J.

[6] Smilowitz NR, Berger JS (2020). Perioperative cardiovascular risk assessment and management for noncardiac surgery: a review. JAMA.

[7] Ghadri JR, Fiechter M, Veraguth K, Gebhard C, Pazhenkottil AP, Fuchs TA, Templin C, Gaemperli O, Kaufmann PA (2012). Coronary calcium score as an adjunct to nuclear myocardial perfusion imaging for risk stratification before noncardiac surgery. J Nucl Med: Off Publ Soci Nuclear Med.

[8] Smilowitz NR, Berger JS (2016). Perioperative management to reduce cardiovascular events. Circulation.

[9] Smilowitz NR, Gupta N, Ramakrishna H, Guo Y, Berger JS, Bangalore S (2017). Perioperative major adverse cardiovascular and cerebrovascular events associated with noncardiac surgery. JAMA Cardiol.

[10] Joshi H, Shah R, Prajapati J, Bhangdiya V, Shah J, Kandre Y, Shah K (2016). Diagnostic accuracy of computed tomography angiography as compared to conventional angiography in patients undergoing noncoronary cardiac surgery. Heart Views: Off J Gulf Heart Assoc.

[11] Nicol ED, Norgaard BL, Blanke P, Ahmadi A, Weir-McCall J, Horvat PM, Han K, Bax JJ, Leipsic J (2019). The future of cardiovascular computed tomography: advanced analytics and clinical insights. JACC Cardiovasc Imaging.

[12] Pflederer T, Marwan M, Schepis T, Ropers D, Seltmann M, Muschiol G, Daniel WG, Achenbach S (2010). Characterization of culprit lesions in acute coronary syndromes using coronary dual-source CT angiography. Atherosclerosis.

[13] Bossone E, Cademartiri F, AlSergani H, Chianese S, Mehta R, Capone V, Ruotolo C, Tarrar IH, Frangiosa A, Vriz O (2021). Preoperative assessment and management of cardiovascular risk in patients undergoing non-cardiac surgery: implementing a systematic stepwise approach during the covid-19 pandemic era. J Cardiovasc Develop Disease.

[14] van Rosendael AR, Bax JJ (2017). Improved risk stratification with computed tomographic coronary angiography in patients with suspected coronary artery disease. Eur Heart J Cardiovasc Imaging.

[15] Abbara S, Blanke P, Maroules CD, Cheezum M, Choi AD, Han BK, Marwan M, Naoum C, Norgaard BL, Rubinshtein R (2016). SCCT guidelines for the performance and acquisition of coronary computed tomographic angiography: a report of the society of Cardiovascular Computed Tomography Guidelines Committee: Endorsed by the North American Society for Cardiovascular Imaging (NASCI). J Cardiovasc Comput Tomogr.

[16] Fleisher LA, Fleischmann KE, Auerbach AD, Barnason SA, Beckman JA, Bozkurt B, Davila-Roman VG, Gerhard-Herman MD, Holly TA, Kane GC (2014). 2014 ACC/AHA guideline on perioperative cardiovascular evaluation and management of patients undergoing noncardiac surgery: executive summary: a report of the American College of Cardiology/American Heart Association Task Force on Practice Guidelines. Circulation.

[17] Chou R (2015). Cardiac screening with electrocardiography, stress echocardiography, or myocardial perfusion imaging: advice for high-value care from the American College of Physicians. Ann Intern Med.

[18] Kofoed KF, Engstrøm T, Sigvardsen PE, Linde JJ, Torp-Pedersen C, de Knegt M, Hansen PR, Fritz-Hansen T, Bech J, Heitmann M (2021). Prognostic value of coronary CT angiography in patients with non-ST-segment elevation acute coronary syndromes. J Am Coll Cardiol.

[19] Pontone G, Bertella E, Mushtaq S, Loguercio M, Cortinovis S, Baggiano A, Conte E, Annoni A, Formenti A, Beltrama V (2014). Coronary artery disease: diagnostic accuracy of CT coronary angiography—A comparison of high and standard spatial resolution scanning. Radiology.

[20] Cohen MC, Aretz TH (1999). Histological analysis of coronary artery lesions in fatal postoperative myocardial infarction. Cardiovasc Pathol.

[21] Wexler L, Brundage B, Crouse J, Detrano R, Fuster V, Maddahi J, Rumberger J, Stanford W, White R, Taubert K (1996). Coronary artery calcification: pathophysiology, epidemiology, imaging methods, and clinical implications. A statement for health professionals from the American Heart Association. Writing Group. Circulation.

[22] Budoff MJ, Shaw LJ, Liu ST, Weinstein SR, Mosler TP, Tseng PH, Flores FR, Callister TQ, Raggi P, Berman DS (2007). Long-term prognosis associated with coronary calcification: observations from a registry of 25,253 patients. J Am Coll Cardiol.

[23] Valenti V, Hartaigh B, Cho I, Schulman-Marcus J, Gransar H, Heo R, Truong QA, Shaw LJ, Knapper J, Kelkar AA (2016). Absence of coronary artery calcium identifies asymptomatic diabetic individuals at low near-term but not long-term risk of mortality: a 15-year follow-up study of 9715 patients. Circ Cardiovasc Imaging.

[24] Chaikriangkrai K, Jhun HY (2017). Palamaner Subash Shantha G, Bin Abdulhak A, Sigurdsson G, Nabi F, Mahmarian JJ, Chang SM. Coronary artery calcium score as a predictor for incident stroke: Systematic review and meta-analysis. Int J Cardiol.

[25] Arnett DK, Blumenthal RS, Albert MA, Buroker AB, Goldberger ZD, Hahn EJ, Himmelfarb CD, Khera A, Lloyd-Jones D, McEvoy JW (2019). 2019 ACC/AHA guideline on the primary prevention of cardiovascular disease: a report of the American College of Cardiology/American Heart Association Task Force on Clinical Practice Guidelines. Circulation.

[26] Lehmann N, Erbel R, Mahabadi AA, Rauwolf M, Möhlenkamp S, Moebus S, Kälsch H, Budde T, Schmermund A, Stang A (2018). Value of progression of coronary artery calcification for risk prediction of coronary and cardiovascular events: result of the HNR Study (Heinz Nixdorf Recall). Circulation.

[27] Shen YW, Wu YJ, Hung YC, Hsiao CC, Chan SH, Mar GY, Wu MT, Wu FZ (2020). Natural course of coronary artery calcium progression in Asian population with an initial score of zero. BMC Cardiovasc Disord.

[28] Hansen PW, Gislason GH, Jørgensen ME, Køber L, Jensen PF, Torp-Pedersen C, Andersson C (2016). Influence of age on perioperative major adverse cardiovascular events and mortality risks in elective non-cardiac surgery. Eur J Intern Med.

[29] Sunny JC, Kumar D, Kotekar N, Desai N (2018). Incidence and predictors of perioperative myocardial infarction in patients undergoing non-cardiac surgery in a tertiary care hospital. Indian Heart J.

